# Dr. Tripler

**Published:** 1861-07

**Authors:** 


DR. TRIPLER.—This gentleman, recently stationed in this city, and so well known as one of the ablest surgeons in the United States Army, is now a member of General Patterson's staff *en route* to Harper's Ferry. Dr. Tripler saw much military practice in Mexico, and has recently published, in conjunction with Dr. Blackman, of Cincinnati, a small work on Military Surgery.

